# The Role of Age and Gender in Perceived Vulnerability to Infectious Diseases

**DOI:** 10.3390/ijerph17020485

**Published:** 2020-01-11

**Authors:** Amelia Díaz, Ángela Beleña, Jesús Zueco

**Affiliations:** 1 Department of Personality, Assessment and Psychological Treatment, Faculty of Psychology, University of Valencia, 46010 Valencia, Spain; Mangeles.belena@uv.es; 2 Department of Microbiology and Ecology, Faculty of Pharmacy, University of Valencia, 46010 Valencia, Spain; Jesus.zueco@uv.es

**Keywords:** age, gender, perceived infectability, germ aversion, biological immune system, behavioral immune system, integrated compensatory immune system

## Abstract

*Background*: The study of the immune system has been approached using two separate paths, the biological immune system and the behavioral immune system. Recently, Gangestad and Grebe proposed a unique integrated compensatory immune system, where both systems work together and one of them could compensate for the other when necessary. However, few studies have confirmed the existence of this integrated compensatory immune system. Our study represents an attempt to explore the existence of this unique immune system, investigating if the behavioral immune system variables increase when the biological immune system weakens with age. *Material and Methods.* The cross-sectional design study was made up of a final sample of 1108 participants (45.2% men and 54.2 women) aged 18–64 years. The younger group (18–21 years) was made up of students, whilst the older groups (22 to 64 years) were composed by their relatives and acquaintances, following the snow ball process. The participants completed the Perceived Vulnerability to Disease Questionnaire that assesses perceived infectability and germ aversion. Correlations, analyses of variance (ANOVAs), and independent group comparisons were performed. These analyses showed the relationships between the variables studied, the effects of age and gender in perceived infectability and germ aversion, and the differences that perceived infectability and germ aversion presented in different age-groups separated by gender. *Results*: A pattern emerged where germ aversion increases as both men and women get older, but perceived infectability decreases up to the age of 50, and then it increases in women from that age onward. Gender differences are only significant in younger participants, with women having higher scores than men in both variables. *Conclusion:* The results partially support the existence of a unique integrated compensatory biological/behavioral immune system.

## 1. Introduction

The study of infectious diseases has included social, behavioral, economic, anatomical, hormonal, and genetic variables as factors affecting these diseases. Therefore, due to the features of these factors, researchers studying the immune system in relation to infectious diseases have taken two diverging paths according to the importance given to the biological or behavioral variables. Both have been relevant and complementary in the study of what protects us from these types of diseases, giving rise to the separation of two immunological systems, a reactive biological system that works mainly when the infection has already occurred and a behavioral system with a more proactive function: avoiding the situations or elements that can lead to contagion or infection [1]. Throughout the history of humankind, infectious diseases have caused serious pandemics that have killed millions of people. Examples dramatically illustrating this point are the “Pandemic Influenza” in 1918 or “Spanish Flu” which is deemed to have killed 20–50 million people [2], or the most recent AIDS pandemic that is estimated to have killed some 35 million people since it was first detected in 1981 [3]. Consequently, the study of both approaches, biological and behavioral, is vital, the first to fight the infectious disease responsible for the pandemic, and the second, to prevent and avoid contagion.

There is a trend in the study of pathogen avoidance that postulates the existence of an integrated compensatory immune system [4], rather than two separate immune systems, biological and behavioral. From the different arguments that justify the existence of this integrated immune system, the compensatory process between the behavioral and the biological systems, illustrated by Fleischman and Fessler’s study [5] could be highlighted. Fleischman and Fessler proposed “the compensatory behavioral prophylaxis hypothesis” based on the fact that women avoid contaminants during the luteal phase and pregnancy when progesterone modulates women´s immune response to tolerate a foreign body. Therefore, the lower functioning of the biological immune system would be compensated by higher levels of infection risk avoidance from the behavioral immune system.

Gender and age separately produce differences in both biological and behavioral immunological systems. Men/women and younger/older comparisons present different prevalence in several infectious diseases and different immunological responses to infectious diseases and vaccines. In addition, gender and age differences happen in variables associated to the behavioral immunological system as perceived infectability, germ aversion, and disgust. Studies performed in recent decades have found that our biological immune system responses are impaired with age [6,7,8,9,10,11,12]. Elderly patients are a good example of this, showing poor vaccine responses and general increased mortality as a consequence of influenza. While 90% of young people respond to vaccination, only 40%–50% of people over 60 do the same [8]. Commonly, the diminished response of the biological immune system with age is attributed to thymic involution and the ensuing decreased production of T cells (responsible for immune responses). This leads to an increased risk of infectious diseases as we get older [9]. Evidence suggests that although the biological immune system weakens at a constant rate with age, there is a pivotal age in the mid- to late fifties (aged 56.3–60.5) that is crucial for screening and intervention [13].

As a general rule, women exhibit a more robust biological immune response to infection than men [14,15,16,17]. Firstly, based on their differences in responses to vaccines, women develop higher antibody responses and greater vaccine efficacy than men [15]. Secondly, sex hormones play a role in the regulation of the transcription of many genes involved in the development and maturation of immune cells, regulation of immune responses, and modulation of immune signaling pathways [16]. Nonetheless, women are not less susceptible to all infectious diseases, being more prone to infections such as Human Immunodeficiency Virus (HIV) [18]. On the other hand, men are more susceptible to infectious diseases, such as tuberculosis [19] or parasitic diseases [20].

In the case of the behavioral immune system, three variables are the most commonly studied: perceived infectability, germ aversion, and pathogen disgust. Pathogen disgust and germ aversion assess emotional responses to stimuli relevant to disease transmission, whereas perceived infectability measures beliefs about perceived immunological functioning and susceptibility to infectious diseases [21]. The first two variables, as affective or emotional responses, could be more specific to a domain of disease-threat, while perceived infectability, as a self-reflective appraisal of vulnerability, could be more general in the domains of disease-threat. Gender differences have been shown for the three variables, with women scoring higher than men, but the effect of age has been studied in pathogen disgust only. The influence of age over disgust sensitivity has been much more modest than that of gender, although it has been commonly found that disgust sensitivity decreases with age [22,23,24]. However, some of these studies were performed with undergraduate students whose age range was very limited. Only specific stimuli were assessed, such as face masculinity and its relationship to pathogen disgust exclusively in young adult women [22], or death-related disgust in women [23]. Hence, the logical result should be the opposite: If aging is associated to a heightened threat of disease, and the biological immune system shows a lower response rate when aging, disgust sensitivity should be higher. The “unexpected” downward trend of disgust sensitivity with age can be explained—firstly, based on the more extensive exposure to experiences of disgust in older people, leading to habituation [24], and secondly given the amount of energy required for the elderly to maintain a high defensive behavior [25].

Gender as a demographic variable has a strong effect on disgust sensitivity in several studies, with women showing more disgust than men [22,26,27,28]. A similar pattern has been shown by perceived infectability and germ aversion, from the Perceived Vulnerability to Disease Questionnaire (PVDQ), with higher scores presented by women rather than men [22,29,30]. The reason for these gender differences has been attributed to at least two reasons. The first is related to fitness shown by men, and higher risk behaviors undertaken, with the subsequent indifference for disease signaling cues that would explain the lower disgust sensitivity in males [25,31]. The second reason is that women would be more prepared to avoid disease threats than men because of the higher investment of energy in raising children. This leads women to take on the role of infectious disease protectors, for themselves and their offspring [23,32].

From the background presented above, it can be assumed that age and gender have an important role in both biological and behavioral immune systems, or alternatively in the integrated compensatory immunological system. Therefore, gender differences are only clear in undergraduates or young people in perceived infectability and germ aversion; and few studies associated age with pathogen disgust. To our knowledge, there are no studies measuring the effect of age separately, or combined with gender, on the specific perceived vulnerability to disease variables, perceived infectability and germ aversion. Thus, there seems to be a significant gap in research on the role of gender in older aged samples, and the effect that age could have on these variables.

Accordingly, it could be hypothesized that the effect of gender on older aged samples would be similar to that presented in undergraduates, with women showing higher perceived infectability and germ aversion than men. On the other hand, based on the compensatory behavioral prophylaxis hypothesis” [5], we could hypothesize that perceived infectability and germ aversion would be higher in older age samples in order to compensate for the natural decrease of efficiency in the biological immune system; thus proving the existence of an integrated compensatory immune system.

## 2. Materials and Methods

### 2.1. Participants

The initial sample consisted of 1135 participants. From these, 27 (2.38%) were excluded from further analyses because of incomplete or insufficient information. The final sample consisted of 1108 participants with an age range of 18 to 64 years (mean age = 33.80 *±* 13.65 years), 501 men (45.2%; mean age = 35.18 ± 13.72) and 607 women (54.8%; mean age = 32.65 ± 13.50). It was made up of undergraduate students in the younger group and their relatives and acquaintances, following the snow ball process, in the older groups. Participants were given the scale and clear instructions on how to fill it out. They completed the scale individually or in small groups. Following previous procedures on the PVDQ, the data collection was performed in the Spring and Autumn of 2018. This was, in an attempt to avoid winter, a period with a higher prevalence of colds, flu, pharyngitis, bronchitis, and other more serious respiratory infections such as pneumonia, which could affect the results. These respiratory infections are more common in winter due to several factors, highlighting the contact with other people in closed spaces, less ventilation of homes or sudden changes in temperature [33]. All participants signed informed consent documents, and feedback was given to the participants after correcting the scale. Participants completed the scale voluntarily, and no money or credits were given in exchange for their collaboration. The study was conducted in accordance with the Declaration of Helsinki, and the protocol was approved by the Ethics Committee of the University of Valencia (20160202).

### 2.2. Inclusion and Exclusion Criteria for Participation

The participants included in the study were aged between 18 and 64 years. Participants were excluded for participation when they reported symptoms that could be attributed to an infectious disease at the time of data collection, since this attribution could affect the participants´ responses to the PVDQ, even in the cases where symptoms could be derived from a non-infectious disease.

### 2.3. Instruments

The Spanish validated version of the PVDQ [34] was completed—a 13-item self-report on a 7-point scale response (with endpoints labelled as “strongly disagree” and “strongly agree”) that measures two factors: perceived infectability, assessed by 7 items (example: “In general, I am very susceptible to colds, flu and other infectious diseases”), and germ aversion, assessed by 6 items (example: “It really bothers me when people sneeze without covering their mouths”). The internal consistency (Cronbach´s alpha) of these subscales in this study was 0.79 for perceived infectability and 0.59 for germ aversion. The germ aversion variable is composed of a list of threatening infectious situations, and the subscale´s internal consistency obtained here is similar to that presented in previous studies: α = 0.61 in Duncan et al. [21]; α = 0.56 in Prokop and Fančovičová [35]; and α = 0.55 in Wu and Chang [36]. Additionally, participants completed a sociodemographic record including age and gender information.

### 2.4. Research Design and Statistical Analyses

The study presents a cross-sectional design [37] that includes age-groups from 18 to 64 years taking into account gender.

Data were analyzed using IBM Corp. Released 2015. IBM SPSS Statistics for Windows, Version 23.0. Armonk, NY: IBM Corp. Frequencies, percentages, mean age, and standard deviation age were obtained for the whole sample, and separately for men and women. Correlations, two-way ANOVAs, and Chi-square tests were performed to find out the relationship between all variables studied and the effects of age and gender on perceived infectability and germ aversion, respectively. To analyze differences between groups in a more detailed way, Bonferroni correction, Student´s *t*, and Cohen´s *d* were performed using independent gender and age-groups. As stated in the introduction, the first age-group should correspond to undergraduates, since most of the studies performed on perceived infectability and germ aversion have been carried out on this segment, so the age-range for this group was 18 to 21. However, the question is how to split the remaining participants into different age-groups in the absence of clear theoretical or biological criteria. Statistical considerations point towards groups of a minimum of 100 participants and a similar number of years in the interval. Furthermore, clarity in the interpretation of results, in order to confirm that they are not the consequence of the grouping chosen in the study, point toward the presentation of different bundles of participants. Following these considerations, the remaining participants were bundled in different ways: as a single group age-ranged 22 to 64; in two groups, age-ranged 22 to 43 and 44 to 64; or three groups age-ranged 22 to 35, 36 to 49, and 50 to 64.

## 3. Results

Pearson correlations were performed for all variables except gender where, due to the categorical nature of the variable, Spearman correlations were obtained. The results show that perceived infectability was significantly related with germ aversion (*r* = 0.15; *p* < 0.001), age (*r* = -0.21; *p* < 0.001), and gender (*r* = 0.17; *p* < 0.001). Germ aversion was also significantly related to age (*r* = 0.30: *p* < 0.001) and gender (*r* = 0.06; *p* < 0.05). Accordingly, perceived infectability and germ aversion were positive and significantly related, whilst perceived infectability showed a negative relationship with age but positive with gender. Germ aversion was positively associated with both gender and age.

In the next stage of the analysis, two-way ANOVAs were performed to examine the effects of gender and age on perceived infectability and germ aversion. The analysis showed significant effects of age and gender in both variables. The main effects of age (*F*_(46.1016)_ = 2.48; *p* < 0.001; partial *ɳ*^2^ = 0.101) and gender (*F*_(1.1016)_ = 4.46; *p* = 0.035; partial *ɳ*^2^ = 0.004) were significant on perceived infectability, as well as the interaction between age and gender (*F*_(44.1016)_ = 1.85; *p* = 0.001; partial *ɳ*^2^ = 0.074). Further, the main effects of age (*F*_(46.1016)_ = 3.52; *p* < 0.001; partial *ɳ*^2^ = 0.137) and gender (*F*_(1.1016)_ = 5.32; *p* = 0.021; partial *ɳ*^2^ = 0.005) were significant on germ aversion but not the interaction between gender and age (*F*_(44.1016)_ = 1.08; *p* = 0.344; partial *ɳ*^2^ = 0.044).

Chi-square tests of independence were performed to examine the relation between gender and age-groups. The relation was not significant (*χ*^2^(3, *n* = 1108) = 7.17; *p* < 0.07) with four age-groups, neither was it significant with three age-groups (*χ*^2^(2, *n* = 1108) = 5.63; *p* < 0.06), but it was significant when two age-groups were analyzed (*χ*^2^(1, *n* = 1108) = 4.45 *p* < 0.04).

The Bonferroni correction in Table 1 presented significant differences in perceived infectability between the younger group (18–21years) and the remaining age-groups, and between the 22–35 and the 36–49 year-groups. Participants in the 50–64 year-group did not present significant differences with the 22–35 and 36–49 year-groups, neither when the comparison were between 22–43 and 44–64 year-groups analyzing three groups. In the case of the variable germ aversion, the differences were high and significant between all age-groups. The younger group presents the lowest values, with values progressively increasing in older groups. For the two age-groups comparison, Student’s *t* test was performed, the younger group (18–21 years) being again the one that presented significantly more perceived infectability (*M* = 24.24 ± 8.06) and lower germ aversion (*M* = 18.22 ± 6.02) than the older one (22–64 years) (*M* = 20.50 ± 7.69), *t*(1106) = 7.11, *p* < 0.001; *d* = 0.48 for perceived infectability, and (*M* = 21.21 ± 6.33), *t*(1106)= −7.07, *p* < 0.001; *d* = 0.048 for germ aversion.

Student´s *t* and Cohen´s *d* were performed, taking into account the gender variable first in the whole sample and then in each age-group. Men and women showed significant differences in both perceived infectability and germ aversion in the whole sample. Perceived infectability was higher in women (*M* = 22.79 ± 8.40) than in men (*M* = 19.98 ± 7.11), *t*(1106) = 6.04, *p* < 0.001; *d* = 0.36. In a similar way, germ aversion was higher in women (*M* = 20.74 ± 6.59) than men (*M* = 19.67± 6.11), *t*(1106) = 2.01, *p* < 0.05; *d* = 0.17. However, these significant differences were not present in all age-groups, as shown in Table 2, where only women between 18 and 21 years old presented higher perceived infectability and germ aversion than men. Perceived infectability was also higher in women than men in the age-groups 22–64, 22–43, and 22–35, but from the four age-groups comparison, it seems that the difference was found in the last group, 22 to 35 years. The difference between men and women is noteworthy in perceived infectability in the age range of 18 to 21 years old, highly significant and with a large effect size.

**Figure 1:**
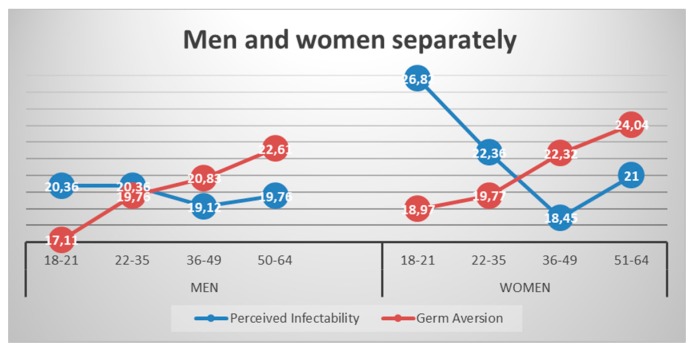
Perceived infectability and germ aversion for the four age-groups (18–21; 22–35; 36–49; and 50–64) in men and women separately.

The representation of the mean in the four age-groups in men and women in Figure 1 clearly shows the important gender differences at the ages of 18 to 35 in perceived infectability. Similar scores are shown at the ages of 36 to 49, where both men and women get the lowest value, their perceived infectability increasing again at the age of 50–64. At this stage, women present more extreme values than men, whose representation line is flatter. Germ aversion shows a similar pattern in both genders. The only peculiarity is that women present higher values than men in all age-groups except at the age of 22 to 35, where their values are similar. At a more specific level, when looking at the germ aversion items, there is a constant increase in the four age groups in the six items or behaviors that made up the factor, with the lowest scores in the 18–21 age-group and the highest in the 50–64 age-group.

## 4. Discussion

Taking our sample as a whole, our results present a picture where women obtained higher perceived infectability and germ aversion scores than men, confirming previous studies with these variables [21,29,30] and with disgust variables [22,26,27,28]. However, a closer look at the results obtained with the sample divided by age-groups reduces these similarities concerning previous results to younger age groups, especially those including undergraduates. Men and women aged over 35 years did not present significant differences in perceived infectability and germ aversion.

The explanation for these gender differences also applies better to younger age-groups than older ones. In a behavioral context, men are more into fitness, health risk activities, and sensation seeking, being more indifferent to infectious and contagion signs than women [25,31]. The peak point in health risk behaviors, such as extreme sport, alcohol and drug consumption, driving, and sexual activities undertaken by men has been suggested to correspond to an age of 35 [38], whilst sensation seeking has been suggested to peak in the twenties [39]. On the other hand, women are more concerned about infectious or contagious clues when they are of child-bearing age, pregnant or raising young children, which occurs in younger women [23,30]. The fact that most studies about gender differences have been performed on samples of young adults may explain why these differences were not so obvious in studies where the sample included a wider range of ages [33].

The results from this study are also more coherent with the biological immune system responses than previous studies using disgust variables in relation to age [22,23,24]. In general, women and young people present a stronger immune response than men and older people [14,15,16,17], but the inflexion point in the biological immune system seems to occur in the fifties, where the necessity for health assessment and screening is especially relevant [13]. Therefore, it may explain why people up to the age of 49 perceive that their immune systems are protecting them, but this perception changes from the ages of 50 to 64 where it goes in the opposite direction. Our results show that this is exactly the evolution that perceived infectability follows in women, dropping until they reach the age of 50, only to increase again from this point onward while the biological immune system is weakening. The differences between men and women in perceived infectability could be tentatively explained by sex hormones. Menopause in women produces an increment in the risk of several diseases, including coronary heart disease [40], osteoporosis [41], and type 2 diabetes [42]. Although all the diseases cited are not infectious, women’s health after menopause seems to be poorer, so they could perceive that their immune system is weaker than before menopause.

The role played by germ aversion in our study is totally concordant with what could be expected from the evolution of the biological immune system, showing an increasing aversion to germs as our own biological immune system weakens with age. Accordingly, germ aversion may be an adequate variable for the study of the integrated compensatory immune system response, as it seems to work in a logical manner: increasing with age to compensate for the progressive weakening of the biological immune system in both genders. Therefore, our study suggests a link between the biological and the behavioral immune systems, where germ aversion in both men and women, and perceived infectability in women over 50 years of age confirm “the compensatory behavioral prophylaxis hypothesis” [5] and partially support the existence of an integrated immune system [4]. Furthermore, our results represent a significant step forward in two of the main issues in the study of the behavioral immune system proposed by Ackeman et al. [43], namely, the linking the biological and behavioral immune systems and the study of the evolution of the behavioral immune system throughout life.

The PVDQ used in this study [34] includes both statements on the resistance or weakness of our immune system, together with others on avoidance or non-avoidance behavior towards germ-risk situations, placing the person being assessed in a situation that goes beyond simple disgust, where the risk of contagion could be high. Sentences related to the participants’ history of susceptibility to infectious diseases, and more specifically, being more likely to catch a cold, flu or other infectious diseases, are combined with sentences on avoiding people sneezing without covering their mouths or washing their hands after touching something that could be contaminated. This combination of statements assumes an awareness of the diversity of bacteria, viruses, and parasites and their possible transmission, in any of the situations listed as items on the scale. Likewise, the relationship between the two PVDQ variables, perceived infectability and germ aversion, significant but low in the studies published [21,34], does not allow us to conclude that a high perceived infectability directly implies a high germ aversion. Mediational variables may affect this relationship in the same way that they could have an important role in the differences presented by men and women in the younger groups in both variables, perceived infectability and germ aversion. Sensation seeking and risky sexual behavior in men, or child bearing age in women may mediate/modulate these differences, and they should be taken into account in future research on the subject. The interpretation of the results of this study is partially limited by the possibility of unmeasured variables having an effect on the results we have found. Cohort/generational effects due to cross-sectional method; demographic variables such as parenthood; and psychological variables such as anxiety and physiological variables such as estrogen levels could all have a role in explaining gender and age effects in perceived infectability and germ aversion. Future research should include these variables in order to discard or confirm this possibility.

Finally, the results should be placed in the specific context from where the sample has been obtained, Spain. Vaccination coverage is 97% (poliomyelitis, diphtheria, tetanus and pertussis, hepatitis B, meningococcal group B and C, pneumococcus, *Haemophilus influenzae* type b, measles, rubella, mumps, and human papillomaviruses) although vaccination is not mandatory. This percentage decreases to 92% in booster doses. It is also significant that some 54% of adults over 64 years were vaccinated against influenza in the winter of 2018–2019 [44]. Therefore, we could conclude that awareness of infectious diseases and confidence on the preventive value of vaccination is relatively high in the Spanish population.

## 5. Conclusions

Our results on the effect of gender and age in perceived vulnerability to infectious diseases variables suggest that gender plays a role in young people only, where women presented higher perceived infectability and germ aversion than men. Germ aversion increased with age in both men and women; however, perceived infectability did not change with age in men, whilst in women, it decreased up to the age of 50 to increase again from this point onward. Overall, the lowered effectiveness of the biological immune system with age seems to be compensated by behavioral variables, mainly germ aversion in men and women, and perceived infectability in women. Accordingly, this study represents a contribution supporting the existence of a unique integrated compensatory biological/behavioral immune system.

## Figures and Tables

**Table 1:** Bonferroni correction with 4 age-groups (left) and 3 age-groups (right).

	Age-groups Comparison		Mean D	*SE*	*p*	Age-groups Comparison		Mean D	*SE*	*p*
Perceived Infectability	18–21	22–35	2.76	0.58	< 0.001	18–21	22–43	3.39	0.57	< 0.001
		36–49	5.45	0.69	< 0.001		44–64	4.29	0.63	< 0.001
		50–64	3.86	0.67	< 0.001					
	22–35	36–49	2.69	0.62	< 0.001					
		50–64	1.10	0.64	0.510	22-43	44–64	0.90	0.56	0.326
	36–49	50–64	−1.60	0.73	0.177					
Germ Aversion	18-21	22–35	−1.54	0.47	0.006	18–21	22–43	−1.94	0.45	< 0.001
		36–49	−3.36	0.56	< 0.001		44–64	−4.61	0.49	< 0.001
		50–64	−5.10	0.54	< 0.001					
	22–35	36–49	−1.81	0.53	0.004	22–43	44–64	−2.67	0.44	< 0.001
		50–64	−3.54	0.51	< 0.001					
	36–49	50–64	−1.74	0.59	0.002					

*SE* = Standard Error; D = Difference.

**Table 2:** Mean differences between men and women in each age-group.

			Perceived Infectability				Germ Aversion			
Age-Groups		n	*M*	*SD*	*t*	*d*	*M*	*SD*	*t*	*d*
18–21	**♂**	121	20.35	6.87			17.11	5.20		
	**♀**	181	26.83	7.76	−7.44 ***	−0.88	18.97	6.42	−2.77 **	−0.32
22–35	**♂**	168	20.36	7.42			19.76	5.86		
	**♀**	213	22.36	8.65	−2.43 *	−0.25	19.77	6.25	0.02	0.00
36–49	**♂**	100	19.12	7.70			20.83	6.26		
	**♀**	101	18.44	6.59	0.67	0.10	22.32	6.28	−1.68	−0.24
50–64	**♂**	112	19.76	6.34			22.61	5.97		
	**♀**	112	21.00	7.62	−1.33	−0.18	24.04	6.34	−1.74	−0.23
22–43	**♂**	224	20.01	7.59			19.88	5.97		
	**♀**	267	21.56	8.49	−2.14 *	−0.19	20.40	6.47	−0.91	−0.08
44–64	**♂**	156	19.64	6.59			22.31	6.02		
	**♀**	159	20.25	7.26	−0.78	−0.09	23.34	6.22	−1.50	−0.17
22–64	**♂**	380	19.86	7.19			20.88	6.10		
	**♀**	426	21.07	8.07	−2.25 *	−0.16	21.50	6.53	−1.38	−0.10

**♂** =Men; **♀**= Women; *M* = Mean; *SD* = Standard Deviation; *t* = Student´s *t-test*; *d* = Cohen´s *d*; * *p* < 0.05; ** *p* < 0.01; *** *p* < 0.001.
